# Multi-Sensor Data Fusion Algorithm Based on Trust Degree and Improved Genetics

**DOI:** 10.3390/s19092139

**Published:** 2019-05-08

**Authors:** Guiling Sun, Ziyang Zhang, Bowen Zheng, Yangyang Li

**Affiliations:** School of Electronic Information and Optical Engineering, Nankai University, Tianjin 300350, China; sungl@nankai.edu.cn (G.S.); zhengbwen@mail.nankai.edu.cn (B.Z.); 1120170104@mail.nankai.edu.cn (Y.L.)

**Keywords:** greenhouse, WSNs, data fusion, improved genetic algorithm, trust degree, cubic exponential smoothing

## Abstract

Aiming at the problems of low data fusion precision and poor stability in greenhouse wireless sensor networks (WSNs), a multi-sensor data fusion algorithm based on trust degree and improved genetics is proposed. The original data collected by the sensor nodes are sent to the gateway through the sink node, and data preprocessing based on cubic exponential smoothing is performed at the gateway to eliminate abnormal data and noise data. In fuzzy theory, the range of membership functions is determined, according to this feature, the data fusion algorithm based on exponential trust degree is used to fuse the smooth data to avoid the absolute degree of mutual trust between data. In this paper, we have improved the crossover and mutation operations in the standard genetic algorithm, the variation is separated from the intersection, the chaotic sequence is used to determine the intersection, and the weakest single-point intersection is implemented to improve the convergence accuracy of the algorithm, weaken and avoid jitter problems during optimization. The chaotic sequence is used to mutate multiple genes in the chromosome to avoid premature algorithm maturity. Finally, the improved genetic algorithm is used to optimize the fusion estimation value. The experimental results show that the cubic exponential smoothing can significantly reduce the data fluctuation and improve the stability of the system. Compared with the commonly used data fusion algorithms such as arithmetic average method and adaptive weighting method, the data fusion algorithm based on trust degree and improved genetics has higher fusion precision. At the same time, the execution time of the algorithm is greatly reduced.

## 1. Introduction

Wireless Sensor Networks (WSNs) is a multi-hop network that combine sensor technology, information processing technology, embedded technology, and wireless communication technology. It consists of a large number of wireless sensor nodes that are deployed, monitored, processed, and transmitted in the monitoring area, with characteristics of small size, low cost, self-organizing networks, and massive scale of coverage [1,2,3]. WSNs can effectively monitor environmental information, and has been widely used in smart agriculture, autonomous driving, and military defense, and plays an increasingly important role [4,5,6]. In a WSN-based greenhouse environmental monitoring system, a large number of homogeneous sensor nodes are usually deployed in the sensing area [7], to perform periodic environmental data collection and transmission. On the one hand, WSNs generates a large amount of redundant data while monitoring information; on the other hand, various environmental parameters in the greenhouse are unevenly distributed, which are easily affected by factors such as sensor accuracy, transmission error, environmental noise, and human interference. The measurement results are low in efficiency, and the system is unstable. Therefore, greenhouse data collected by multiple sensors must be fused.

Data fusion, also known as information fusion or multi-sensor data fusion [8], refers to multi-sensor data resources that make full use of different time and space, multi-sensor data obtained through by time series, using computer technology to analyze, synthesize, dominate, and apply under specific criteria, and where a consistent interpretation and description of the measured object is obtained [9], so as to realize the corresponding decision and estimation. The system is able to get more accurate information to achieve the purpose of improving system stability. According to the level of information representation, data fusion can be divided into datalevel fusion, featurelevel fusion, and decisionlevel fusion [10]. Among them, datalevel fusion is also called pixel-level fusion [11]. As the lowest level of fusion, the datalevel fusion directly fuses the collected raw data, then the feature vector is extracted from the merged data and before being judged and recognized. There is no data loss problem, and the obtained result is also the most accurate.

Data fusion is a crucial technology for solving low-precision and poor stability in greenhouse WSNs. From different time and space multi-source data, it eliminates redundant information and reduces data transmission, thus achieving the purpose of improving information collection accuracy and enhancing system stability.

The fuzzy theory was developed on the basis of the fuzzy set theory founded by Prof. LA zadeh of the Department of Electrical Engineering of the University of California, Berkeley in 1965. It mainly includes fuzzy set theory, fuzzy logic, fuzzy reasoning, and fuzzy control.

As early as the 1920s, the famous philosopher and mathematician B. wrote a paper on ambiguity. He believed that all natural languages were vague, such as red and old. The concept has no clear connotation and extension, so it is ambiguous and vague. However, in a specific environment, when people use these concepts to describe a specific object, they can understand the truth and rarely cause misunderstanding and ambiguity.

Prof. LA zadeh of the University of California published a famous paper in 1965. For the first time, he proposed an important concept of expressing the ambiguity of things: membership function, which broke through the classical collection theory of Rene. Descartes in the late 19th century. Laid the foundation of fuzzy theory. In 1966, P.N. Marinos published a research report on fuzzy logic. In 1974, L.A. zadeh published a research report on fuzzy reasoning. Since then, fuzzy theory has become a hot topic. In 1974, the British E.H. Mamdani realized the world’s first experimental steam engine control with fuzzy logic and fuzzy reasoning for the first time, and achieved better results than the traditional direct digital control algorithm, thus proclaiming the birth of fuzzy control. In 1980, Denmark’s L.P. Holmblad and Ostergard used fuzzy control in the cement kiln and achieved success. This is the first commercialized and practical fuzzy controller.

Fuzzy theory refers to the theory that uses the basic concept of fuzzy sets or continuous membership degree functions. There are five main branches of fuzzy theory:

- Fuzzy mathematics, which replaces classical sets with fuzzy sets, thus extending the concepts in classical mathematics;
- Fuzzy logic and artificial intelligence, which introduces approximate reasoning in classical logic, and develops expert systems based on fuzzy information and approximate reasoning;
- Fuzzy system, which contains fuzzy control and fuzzy methods in signal processing and communication;
- Uncertainty and information, which is used to analyze various uncertainties;
- Fuzzy decision, which uses soft constraints to consider optimization problems.

The development of fuzzy theory has been close to more than 50 years, and the scope of application is very wide. From the practical application point of view, the application of fuzzy theory is mostly concentrated on fuzzy systems, especially focusing on fuzzy control. There are also some fuzzy expert systems for medical diagnosis and decision support. Since fuzzy theory is still a new thing from the perspective of theory and practice, we expect that, as the fuzzy field matures, more reliable practical applications will emerge.

In this paper, we propose a data fusion method based an improved genetic algorithm in the data layer, aiming at reducing a large amount of redundant information generated in wireless sensor networks, improving the accuracy of information collection, and enhancing the stability of the system. In the greenhouse environment based on wireless sensor networks, the main contributions of our proposed data fusion scheme are as follows:

- Using the cubic exponential smoothing method, data preprocessing is performed on the raw data collected by the sensor nodes, and the abnormal data generated by various factors are eliminated, and the authenticity and reliability of the data are improved;
- For the processed data, the data fusion algorithm proposed in this paper is used for data-level fusion. On the one hand, setting the trust function to exponential form avoids the absolute degree of mutual trust between data and makes the fusion result more accurate. On the other hand, the crossover and mutation operations in the traditional genetic algorithm are improved, the implementation efficiency of the algorithm is improved, and the data fusion accuracy is further improved, and can meet the requirements of high precision, low power consumption, and real-time performance of information collection in a greenhouse environment based on wireless sensor networks.

In the next section, we will present the research status of data fusion in wireless sensor networks. The third part describes the multi-sensor data fusion structure model. The fourth part introduces the data preprocessing method based on the cubic exponential smoothing method. The fifth part proposes a multi-sensor data fusion algorithm based on trust and improved genetics. The sixth part verifies the quasi-determination and stability of the algorithm through simulation. The seventh part is the conclusion of this paper.

## 2. Related Work

Zhang Yulin et al. [12] adopted an improved BP weight balance algorithm, based on wavelet neural networks to fuse measurement data based on feature level [13], and provided data fusion results to decision and judgment, which improved learning speed and calculation accuracy. However, the structure is complicated, the operation is cumbersome, and the dimensionality disaster is easily generated. Wang Haitao et al. [14] proposed a quadratic data fusion algorithm based on trust degree, which shows certain advantages in the case of extreme data fusion. In the literature [15], the author proposed a data fusion algorithm based on arithmetic means weighting, which has a fast calculation speed but has poor anti-interference ability and low fusion precision. Yager [16] proposed a data fusion algorithm based on support degree function, which does not require prior probability statistical knowledge [17]. Only the sensor data at the current time is needed to calculate the optimal fusion value. The disadvantage is that historical data cannot be used, and low fusion accuracy. Zhang et al. [18] used adaptive weighting method, and Cai Zhenjiang et al. [19] use to the mean-based batch estimation method for data fusion, without any prior knowledge of sensor measurement data, objectively reflecting the reliability of each sensor, fusion accuracy higher. However, the observation error required for sensor variance estimation by adaptive weighting method must be zeromean stationary noise.[20], and improper selection of sensor grouping based on batch estimation will affect the final fusion effect [21]. Kalman filtering has also been widely used in data fusion, but there are many serious problems. On the one hand, the increase in the number of sensor nodes increases the number of faults, and, when a sensor node fails, it will contaminate the final fusion result: in terms of its requirements, the state space model of the system is strict, and the accuracy of the model directly affects the data fusion effect [22,23]. Collotta et al. [24] adopted a data fusion scheme based on fuzzy aggregation theory. Although fusion precision is improved, the method can only aggregate accurate data and cannot process complete data. Ziteng Wen et al. [25] proposed a robust data fusion algorithm for data distortion, data loss, and signal saturation during infrared flame detection. The algorithm combines Radial Basis Function (RBF) neural network and Takagi Sugeno (TS) fuzzy model, and the experimental data collected by the three-channel infrared flame detector is used to verify the robustness of the proposed method. The experiment results show that the convergence rate, accuracy, and generalization ability of the proposed method improved are compared with the traditional RBF neural network with TS fuzzy model in [26] and the GA-BP (Genetic Algorithm BackPropagation model in [27]. D. Xu et al. [28] used genetic algorithms and partial least squares regression (GA-PLSR) to select feature bands to reduce data redundancy and achieve rapid measurement of soil properties. Juan Wu et al. [29] proposed a hybrid data fusion scheme based on a least squares support vector machine (LS-SVM) regression model and an adaptive neural fuzzy inference system (ANFIS) decision model. The experimental results show that the high-precision prediction results make the hybrid fusion scheme a reliable and effective method for intelligent control of tobacco. Aiello et al. [30] used decision-level data fusion algorithms to gather information from wireless sensor networks, and aggregated information from all sensors using most rules to make decisions about the possible risks of pest disease. The experimental results show that, by monitoring climatic conditions, the potential risks of pests can be discovered and how decisions can be made to prevent the spread of pest diseases. Kostas et al. [31] proposed a real-time data fusion mechanism based on multivariate sensor data streams, which are used to aggregate context data streams in context theory while detecting and eliminating outliers. In addition, the time series is used to predict the future aggregated value, and finally the context fusion value and the predicted value are input to the type-2 fuzzy inference system to obtain high-accuracy event recognition. Liu et al. [32] applied a data fusion method to health monitoring systems and developed a new data-level fusion model. The model fuses the information of multiple degraded signals to construct a comprehensive health index, which solves the problem of predicting when multiple sensors simultaneously monitor the health status of degraded units.

Regarding the issue above, this paper firstly uses the cubic smoothing method to preprocess the raw data collected by the sensor nodes. According to the fuzzy set theory, a multi-sensor data fusion algorithm based on exponential trust degree is proposed, combined with the improved genetic algorithm, the fusion model is optimized. By adjusting the weight to reduce the error between the measured value and the real value, the fusion precision is improved, and the multi-sensor data fusion of the greenhouse WSNs environmental monitoring system is realized.

## 3. Multi-Sensor Data Fusion Structure Model

The greenhouse WSNs system consists of the terminal node, sink node, and regional gateway, the networking is completed using a star structure. The terminal node is responsible for collecting sensor measurement information, the sink node mainly undertakes data receiving and forwarding tasks, and the regional gateway implements data exchange with the background server and management of the wireless networks.

**Figure 1:**
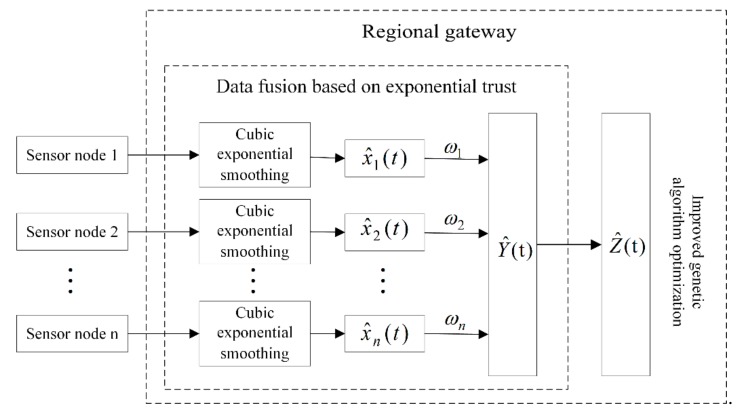
Greenhouse WSNs system data fusion structure model diagram.

The data fusion structure model based on the greenhouse WSNs system is shown in Figure 1. Firstly, the data collected by the sensor nodes is sent to the regional gateway through the sink node, and the original data is smoothed by the cubic exponential smoothing method at the gateway, and the abnormal data and the noise data are eliminated, thereby improving the anti-interference of the system. Datalevel fusion using a trust degree-based data fusion algorithm. According to the defined exponential trust function, the degree of trust between the smoothed data is quantified, and the degree of trust of each smoothed data is measured by the trust matrix to allocate reasonably. The optimal weight $\omega_{i}$ of each sensor node in the fusion process is obtained by the expression of data fusion estimation. If the degree to which a sensor is trusted by other sensors is greater, the the impact of the data collected by this sensor on the fusion results greater. Finally, the improved genetic algorithm is used to optimize the fusion result to further improve the fusion precision, thus achieving multi-sensor data fusion.

## 4. Data Preprocessing Based on Cubic Exponential Smoothing

The greenhouse environment parameters generally change slowly, and the real value of the sensor can be considered to remain unchanged for a short period of time. Data acquisition is subject to sensor accuracy, complex environmental factors, and random faults (such as sensor node damage, energy exhaustion), so the raw data collected is preprocessed.

The traditional data smoothing method mainly adopts a moving average. It is considered that the latest N-phase data has the same influence on the future value, and is weighted by 1/N. The data before the N-phase has no effect on the future value, and the weight is 0. However, the weights of the second and higher moving averages are not 1/N, and the higher the number of times, the more complex the structure of the weights, but the symmetric weights are always maintained, that is, the weights of the two ends are small, and the middle term the weight is large and does not conform to the dynamics of the general system. However, the impact of historical data on the future of the greenhouse environment on future values is decreasing over time. Therefore, the exponential smoothing method is used to process the measured values of each period, and the weighted average is used as the predicted value in chronological order. This data processing method is more practical and has a simple recursive form.

Since the variation of the greenhouse environmental parameters (temperature, humidity, illumination, conductivity, etc.) shows a quadratic curve trend, it is more appropriate to use the cubic exponential smoothing method. The recursive calculation formula is

(1) $$\left\{\begin{array}{c} \;S_{t}^{\left(1\right)}=\;\alpha x_{i}\left(t\right)+\left(1-\alpha \right)S_{t-1}^{\left(1\right)}\\ S_{t}^{\left(2\right)}=\;\alpha S_{t}^{\left(1\right)}+\left(1-\alpha \right)S_{t-1}^{\left(2\right)}\\ S_{t}^{\left(3\right)}=\;\alpha S_{t}^{\left(2\right)}+\left(1-\alpha \right)S_{t-1}^{\left(3\right)} \end{array}\right.,$$

In Formula (1), where α is the weighting coefficient, $0<\alpha <1,\;x_{i}\left(t\right)$ is the data collected by sensor node ***i*** at time *t*, $S_{t}^{\left(1\right)},S_{t}^{\left(2\right)},S_{t}^{\left(3\right)}$ is first smoothing value, a secondary smoothing value, and a cubic smoothing value of the data collected by the sensor ***i*** at time ***t***, respectively. When exponential smoothing is performed, the choice of weighting coefficients is very important. The size of α is proportional to the correction range, and the number of historical data participating in the average is controlled. Considering that the greenhouse environmental data sequence is not very volatile and is relatively stable, the value of α should be set in the range of 0.1 to 0.3 to reduce the correction range, so that the smoothed value contains historical data for a long time. With regard to the determination of the initial value $S_{0}^{\left(1\right)},S_{0}^{\left(2\right)},S_{0}^{\left(3\right)}$ of the Formula (1), when the historical data is large (more than 20), the initial value has little influence on the future predicted values. Therefore, this paper takes the average of the five historical data before the time series as the initial value.

## 5. Greenhouse WSNs Data Fusion Algorithm Based on Trust Degree and Improved Genetics

### 5.1. Trust Degree Function

For the data uncertainty problem in the multi-sensor environmental monitoring system, the degree of credibility of the fused data must be determined first in the data fusion process. It is assumed that a plurality of sensors measures the same parameter, and $x_{i}$ and $x_{j}$ respectively represent the cubic exponentially smoothed data measured by the ***i***-th sensor and the ***j***-th sensor at the same time. If the authenticity of $x_{i}$ is higher, the degree to which $x_{i}$ is trusted by the rest of the data is higher. The so-called $x_{i}$ being trusted by $x_{j}$, that is, from the perspective of $x_{j}$, $x_{i}\;$is the possible degree of real data, and the degree of trust between multi-sensor measured data is called trust degree [33]. 

We further quantify and process the trust degree between smoothed data, a trust degree function $b_{ij}$ is defined to indicate the extent to which $x_{i}$ is trusted by $x_{j}$. According to the definition of trust degree, we get $b_{ij}=f\left(\left|x_{i}-\;x_{j}\right|\right)\;i,j=1,2,\ldots ,n$,  f() is a continuous descending function, 0≤f()≤1.

This paper defines the trust degree function $b_{ij}$ as the form of an exponential function. We define

(2) $$b_{ij}=\;\left\{\begin{array}{c} e^{-\left|x_{i}-x_{j}\right|\;\;\;}\;\;\left|x_{i}-\;x_{j}\right|\leq M\\ \;\;\;\;\;\;\;\;0\;\;\;\;\;\;\;\;\;\left|x_{i}-\;x_{j}\right|>M \end{array}\right..$$

It can be seen from the definition form of the Formula (2) that the smaller the value of $\left|x_{i}-\;x_{j}\right|$ is, the larger the $b_{ij}$ the greater the mutual trust between the data $x_{i}$ and $x_{j}$. Since the exponential function $b_{ij}$ monotonically decreases from 0 to 1 on $\left|x_{i}-\;x_{j}\right|\in \left[0,+\infty \right]$, the property that the trust function should have is satisfied. In practical applications, when the value of $\left|x_{i}-\;x_{j}\right|$ exceeds the set upper limit value M (M > 0), it can be considered that the two data no longer trust each other, and, at this time, $b_{ij}=0$.

In Formula (2), $b_{ij}$ is defined as an exponential function form that satisfies the fuzzy property, which not only makes full use of the advantages of the range of membership function in fuzzy theory, but also avoids the absolute degree of mutual trust between data [34]. It is more in line with the authenticity of the actual problem, making the fusion result more accurate and stable [35].

In this paper, we set n sensors to measure the same parameter at the same time, and the trust matrix B is established according to the trust function $b_{ij}$ between the data.

(3) $$B=\left[\begin{array}{cc} \begin{array}{cc} b_{11} & b_{12}\\ b_{21} & b_{22} \end{array} & \begin{array}{cc} \cdots & b_{1n}\\ \cdots & b_{2n} \end{array}\\ \begin{array}{cc} \vdots & \vdots \\ b_{n1} & b_{n2} \end{array} & \begin{array}{cc} \vdots & \vdots \\ \cdots & b_{nn} \end{array} \end{array}\right]$$

For the ***i***-th row element in B, if the value of $\overset{n}{\underset{j=1}{\sum }}b_{ij}$ is large, it indicates that the ***i***-th smooth data is trusted by most sensors; conversely, the ***i***-th smooth data is less likely to be real data.

### 5.2. Trust Degree based Data Fusion Model

The weight of the ***i***-th smoothed data $x_{i}$ in the fusion process is represented by $w_{i}$. Since the size of $w_{i}$ reflects the comprehensive trust degree of other smooth data pairs $x_{i}$, the weighted sum of $x_{i}$ can be weighted by $w_{i}$ to obtain the expression of data fusion.

(4) $$\hat{Y}=\overset{n}{\underset{i=1}{\sum }}w_{i}x_{i}\;\;\;\;i=1,2,\ldots ,n,$$

In Formula (4), where the weight coefficient $w_{i}$ of $x_{i}$ satisfies $\overset{n}{\underset{i=1}{\sum }}w_{i}=1,0\leq w_{i}\leq 1$.

In the trust degree matrix B, the trust degree function $b_{ij}$ only indicates the degree of trust of $x_{i}$ to $x_{j}$, and does not reflect the degree of trust of all smooth data in the system to $x_{i}$, and the true degree of $x_{i}$ is actually synthesized by $b_{i1},b_{i2},\ldots ,b_{in}$ to reflect. Therefore, $w_{i}$ should synthesize all the information about each subsystem $b_{i1},b_{i2},\ldots ,b_{in}$ in the trust degree system of $x_{i}$, and needs to find a set of non-negative numbers $a_{1},a_{2},\ldots ,a_{n}$, so that

(5) $$w_{i}=a_{1}b_{i1}+a_{2}b_{i2}+a_{n}b_{in}\;\;\;\;i=1,2,\ldots ,n.$$

According to the Formula (3), the Formula (5) is rewritten into a matrix form of W=BA. Where $W={\left[w_{1,}w_{2},\ldots ,w_{n}\right]}^{T},\;A={\left[a_{1},a_{2},\ldots a_{n}\right]}^{T}$. Since $b_{ij}\geq 0$, the trust matrix ***B*** is a non-negative matrix, and there is a maximum eigenvalue *λ (λ > 0)*, which make it satisfy

(6) λA=BA.

Calculate ***λ*** and the corresponding feature vector ***A***, and satisfy the condition of component $a_{i}>0\left(i=1,2,\ldots ,n\right)$ in *A*, and bring *λA = BA* into W=BA. We get

(7) W=λA.

Formula (7) can be used as a measure of the degree of integrated trust between smoothed data, that is $\frac{w_{i}}{w_{j}}=\frac{a_{i}}{a_{j}},i,j=1,2,\ldots n$. Considering that $w_{i}$ should satisfy the condition that the weighted sum is 1, normalize the $w_{i}$ to obtain

(8) $$w_{i}=\frac{a_{i}}{a_{1}+a_{2}+\cdots +a_{n}}.$$

Bring Formula (8) into Formula (4), and get the final result of all smooth data fusion estimates:

(9) $$\hat{Y}=\frac{\sum_{i=1}^{n}a_{i}x_{i}}{a_{1}+a_{2}+\cdots +a_{n}}.$$

### 5.3. Optimize Fusion Results with Improved Genetic Algorithms

Genetic algorithm (GA) is a global optimal algorithm based on the principle of natural selection and genetic evolution. Use operating such as selection, crossover, and mutation to combine chromosomes to achieve continuous update of chromosomes, follow the principle of, survival of the fittest and evolve from generation to generation, and finally get the optimal solution. The standard genetic algorithm has the following operations: the generation of the initial population, calculate of the fitness of each individual, the selection of right individuals according to the principle of survival of the fittest, the selection of excellent individuals, pairwise matching, through random crossover of their chromosome genes, and random matching after mutating the genes of certain chromosomes, is generated of the next generation population, and, in this way, is evolved of the population from generation to generation until the evolution termination condition is satisfied.

As one of the modern optimization algorithms, a genetic algorithm is characterized by the ability to jump out the optimal local solution with probability 1 for the nonlinear extremum problem, and then find the globally optimal solution, which is based on the intersection and variation in the algorithm. In the structure of traditional genetic algorithms, mutations are carried out on the basis of crossover, emphasizing the cross-action, and thinking that variation is only a biological background mechanism. Crossovers are usually divided into breakpoint intersections, multipoint intersections, and uniform intersections. Interruption point intersections randomly select a breakpoint in the gene sequence and then exchange all chromosomes at the right end of the parental breakpoint. In mutation operations, mutation operators are generally implemented using random variations of the Gaussian distribution [36,37]. In recent years, some scholars have tried to use the random sequence of Cauchy distribution to achieve variation [38], and hope to achieve a broader range of variation through the broad two-wing characteristics of Cauchy distribution, in order to find the optimal global solution [39]. Chellapilla [40] theoretically analyzed the local convergence of the Cauchy distributed random mutation evolution algorithm, and further combined the two, using the linear superposition of the two distributions, but the simulation results show that the algorithm improvement effect is not apparent. Wu Xiangxing et al. [41] regarded biological evolution as randomness plus feedback and pointed out that the randomness is mainly caused by the internal factors of the system, rather than by the random disturbance of the external environment. The chaotic system appears as random in its chaotic domain, which is a reflection of the internal randomness of the system, which is different from the external random characteristics.

Based on the above problems, this paper improves the standard genetic algorithm. The steps of the improved genetic algorithm are as follows:

**Step 1:** Encoding

Using decimal coding, the weights $w_{i}$ in Formula (8) are composed into random numbers $w_{1},w_{2},\ldots ,w_{n}$, and the random number column is taken as a chromosome, where $0<w_{i}<1,\left(i=1,2,\ldots ,n\right),w_{1}=0,w_{n}=1$; each random number column corresponds to an individual in the population. 

**Step 2:** Set the initial population

This paper uses the improved circle algorithm to set a better initial population, i.e., for the initial circle,

$$C=\pi_{1}\cdots \pi_{u-1}\pi_{u}\pi_{u+1}\cdots \pi_{v-1}\pi_{v}\pi_{v+1}\cdots \pi_{n},1\leq u\leq v\leq n,1\leq \pi_{u}\leq \pi_{v}\leq n.$$

Exchange the order of u and v. The new path at this time is:

$$\pi_{1}\cdots \pi_{u-1}\pi_{v}\pi_{v-1}\cdots \pi_{u+1}\pi_{u}\pi_{v+1}\cdots \pi_{n},$$

Recorded as

(10) $$∆f=\left(d_{\pi_{u-1}\pi_{v}}+\;d_{\pi_{u}\pi_{v+1}}\right)-\left(d_{\pi_{u-1}\pi_{u}}+\;d_{\pi_{v}\pi_{v+1}}\right)$$

**Step 3:** Fitness assessment

The absolute value of the error between the fusion value $\hat{Y}$and the real value $\bar{Y\;}$is defined as the objective function, that is,

(11) $$\rho =\left|\bar{Y}-\hat{Y}\right|=\left|\frac{\sum_{i=1}^{n}\hat{x_{i}}}{n}-\frac{\sum_{i=1}^{n}a_{i}x_{i}}{a_{1}+a_{20}+\cdots +a_{n}}\right|.$$

The objective function in Formula (11) is taken as the fitness function. Therefore, it is transformed into the minimum problem of finding ρ.

**Step 4:** Cross operation

In this paper, we adopt an improved crossover. The specific design is as follows: first of all, the parental individual is paired according to the principle of “door-to-door” that is parent sort by the fitness function value. Usually, the objective function is used as the fitness function, and the objective function value is small. An individual with a little objective function value is paired with a small individual, and an individual with a large objective function value is paired with a large individual. The chaotic sequence is then used to determine the location of the intersections, and finally cross the identified cross terms. For example, $\left(x_{1},x_{2}\right)$ pairing, their chromosomes are $x_{1}=\omega_{1}^{1}\omega_{2}^{1}\cdots \omega_{n}^{1}$,$\;x_{2}=\omega_{1}^{2}\omega_{2}^{2}\cdots \omega_{n}^{2}$, using Logistic chaotic sequence $x\left(n+1\right)=4x\left(n\right)\left(1-x\left(n\right)\right)$ produces a positive integer between 1 and n. Specific steps are as follows:

Take a random initial value of (0,1) and use $x\left(n+1\right)=4x\left(n\right)\left(1-x\left(n\right)\right)$ to iterate once to generate a chaotic value on (0,1), and save the above chaos value. The value is used as the initial value of the chaotic iteration to generate the next-generation cross term, and then the initial value is multiplied by the number n of sensor nodes, and finally rounded to obtain the crossover operator $p_{c}$. Obviously, this single-point crossover has little change to the original solution, which can weaken the jitter problem in the optimization process generated by the standard genetic algorithm in practical applications, and further improve the convergence accuracy of the algorithm.

**Step 5:** Variation

Mutation is also a means to achieve group diversity, and it is an essential guarantee for jumping out of local optimum and making global optimization. The mutation operator $p_{m}$ used in this paper is designed as follows: First, according to the given mutation rate (generally, the probability of variation is small, this paper chooses 0.01), randomly select the integer between 1 and n, mutating genes at the corresponding positions of these two numbers. Perform mutations with the current gene value as the initial value, and iterating using the chaotic sequence $x\left(n+1\right)=4x\left(n\right)\left(1-x\left(n\right)\right)$ to get the new gene value after the mutation, thereby obtaining a new chromosome.

**Step 6:** Choose

The purpose of the selection is to select the right individuals from the current group and make them as the parent generation and breed descendants for the next generation. The genetic algorithm reflects Darwin’s survival of the fittest principle through the selection process. This paper sets the number of individuals in each generation group to be equal, and arranges the individuals in the population according to the degree of fitness, and use the roulette Wheel Selection to select. The probability that each individual is selected is proportional to the value of its fitness function value.

After the selection is completed, the above steps 4~6 are repeated, and when the number of iterations is reached, the evolution is terminated, and the optimal estimation value of the objective function is obtained.

The whole process of Algorithm 1 about improving the genetic algorithm following is the:

**Algorithm 1** Improved genetic algorithm.**1.** Initialization: using a decimal coding strategy, using the random number sequence $w_{1},w_{2},\ldots ,w_{n}$ composed of weights $w_{i}$ as the chromosome, the number of iterations *G* = 500;**2.** Set the initial population size using the improved circle algorithm;**3.** Initial circle $C=\pi_{1}\cdots \pi_{u-1}\pi_{u}\pi_{u+1}\cdots \pi_{v-1}\pi_{v}\pi_{v+1}\cdots \pi_{n};$   **while** ∆f<0 do    **if** New path≠Old path **do**     Exchange the order between u and v to get a new path:         
$\;\pi_{1}\cdots \pi_{u-1}\pi_{v}\pi_{v-1}\cdots \pi_{u+1}\pi_{u}\pi_{v+1}\cdots \pi_{n}$
    **else**      Original path
**    end if**
  **end while;****4.** The objective function  ρ is used as a fitness function;
****5.  for****
G≤500
**do;**
**6.  ** Adopt improved crossover:  **sort** Objective function ρ;  The crossover operator $p_{c}$ is determined by using Logistic chaotic sequence
$x\left(n+1\right)=4x\left(n\right)\left(1-x\left(n\right)\right)$
  According to the set mutation rate, the chaotic sequence $x\left(n+1\right)=4x\left(n\right)\left(1-x\left(n\right)\right)$ is used to obtain the new gene value after mutation, thereby obtaining a new chromosome.**7.** Use the "Roulette Wheel Selection" to choose;**8.** **end for.**

This paper mainly improves the crossover and mutation operations in the standard genetic algorithm. Firstly, the variation is separated from the intersection, making it an independent and cross-parallel optimization operation, so that the genetic algorithm can also be realized by parallel computing, Algorithm implementation efficiency. Secondly, crossover and mutation operations with different intensity of change are used. In the process of genetics, respectively chaos link to genetics; in the cross-operation, the individual is paired by the principle of “door-to-door”, the chaotic sequence is used to determine the intersection, and the weakest single-point intersection is implemented to ensure the convergence accuracy of the algorithm, weaken and avoid the chattering problem in the optimization process caused by excessive cross strength; in the mutation operation, chaotic sequences are used to mutate multiple genes in the chromosome to avoid algorithm premature maturity.

## 6. Tentative and Analysis

### 6.1. Tentative Method

**Figure 2:**
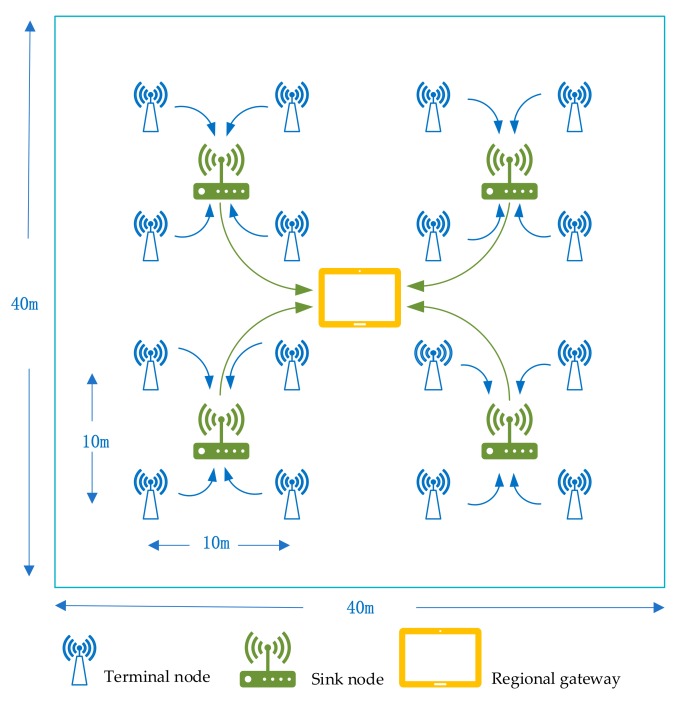
Greenhouse WSNs system topology diagram.

The tentative was carried out on a modern farm where the crops were lettuce. The topological structure of the greenhouse WSN system is shown in Figure 2. The farmland test base is 40 m long and 40 m wide. The clustering method is adopted. 16 temperature and humidity acquisition modules are evenly deployed in the monitoring area as terminal nodes, and one sink node is placed in the center of every four terminal nodes. The regional gateway is arranged at the center of the area, and the height from the ground is 1 m. Considering that the area is relatively small, the star network structure is used for networking. The temperature and humidity acquisition module consists of temperature and humidity composite sensor DHT11 and ZigBee-based wireless transmission module CC2530. CC2530 is also used as the sink node. The gateway use Samsung’s S5P4418 chip as the core processor.

Considering that the environmental data in the greenhouse is changing relatively slow, 16 temperature and humidity nodes collect data every hour. The collection period is from 6:00 to 22:00 on 15 January 2019, and 16 data sets are collected for each node. Through the gateway, the actual data collected by the sensor node, the data after three exponential smoothing processing, and the data after fusion optimization are sent to the background server for storage. Data analysis is performed using MATLAB (R2018a). The configuration of the server is as follows: CPU is Intel(R) Core(TM) i5-7300HQ (2.5 GHz), RAM size is 8 G, and the operating system is Windows10 Professional.

### 6.2. Data Preprocessing Effect

**Figure 3:**
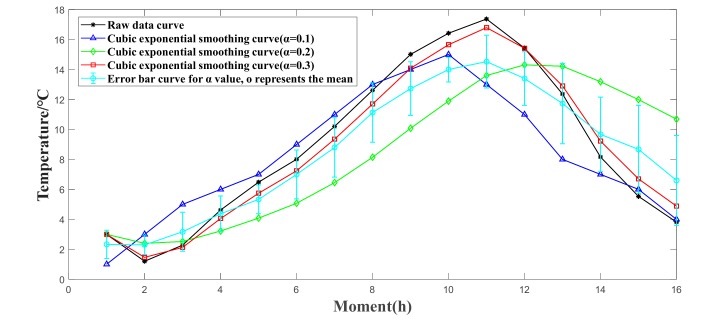
Effect of raw data and three exponential smoothing.

Taking the temperature data collected by a certain temperature and humidity sensor node as an example, Figure 3 is the raw data and the effect after the cubic exponential smoothing (smoothing coefficient α is taken as 0.1, 0.2 and 0.3 respectively), and the error bar of the alpha value. It can be seen that the original data fluctuates greatly, and the temperature curve after three times of cubic smoothing is smoother, and the data fluctuation is small. Within the allowable range of error, the data after the cubic exponential smoothing processes can better represent the original data. When α is 0.2, the smoothing effect is best, but there is an apparent hysteresis deviation; when α is 0.3, the smoothed data better track the trend of data, but the volatility is larger; when α is 0.1, the smoothing effect is better, and the hysteresis is not large, so it is more appropriate to take α 0.1 from.

### 6.3. Data Fusion and Optimization Results

After data preprocessing, 16 sets of smooth temperature data sequences are obtained. Firstly, the degree of trust between the smoothed data $x_{i}$ and $x_{j}$ is calculated by the set upper limit value M, taking M = 0.5. When $\left|x_{i}-\;x_{j}\right|\geq 0.5$, it is considered that the two data no longer trust each other, at this time $\;b_{ij}\;$ = 0. Thus, a 16 × 16 trust degree matrix *B* is obtained, and 16 sets of smoothed data at the same time (Here, we take 6:00) are calculated according to Formula (6), thus the maximum eigenvalues of the trust degree matrix of 16 sets of smoothed data and the corresponding feature vector are obtained.

λ=13.2342

*A* = [ 0.2597, 0.2857, 0.2183, 0.2690, 0.2453, 0.2732, 0.2335, 0.2686, 0.2291, 0.2622, 0.2208, 0.2465, 0.2367, 0.2732, 0.2324, 0.2326]

The comprehensive support degree of the first set of data is obtained by Formula (8).

*W* = [ 0.0651, 0.0717, 0.0548, 0.0675, 0.0615, 0.0685, 0.0586, 0.0674, 0.0575, 0.0658, 0.0554, 0.0618, 0.0594, 0.0685, 0.0583, 0.0583]

In the same way, the comprehensive support degree of the remaining 15 sets of smoothed data is calculated, and all of them are verified to satisfy the condition of $\overset{n}{\underset{i=1}{\sum }}w_{i}=1$.

Finally, the genetic algorithm improved by this paper is used to optimize the fusion result, reduce the error between the fusion estimation value and the real value, and bring the obtained weight W into Formula (11) to get the fitness function, that is, the objective function. 

(12) $$\rho =\left|\bar{Y}-\hat{Y}\right|=\left|\frac{\sum_{i=1}^{n}\hat{x_{i}}}{n}-\frac{\sum_{i=1}^{n}a_{i}x_{i}}{0.0651+0.0717+\cdots +0.0583}\right|.$$

**Figure 4:**
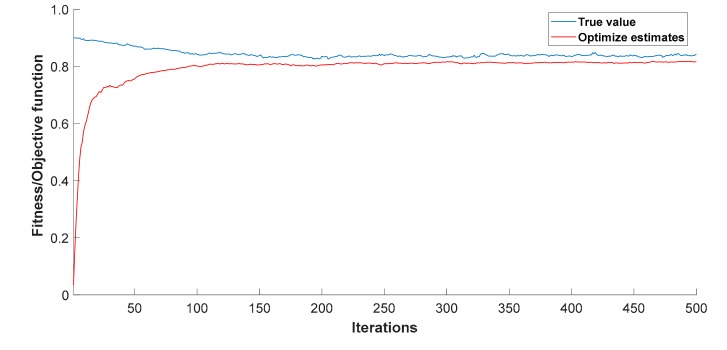
Optimized fitness curve.

Firstly, the initial population size is set to 50, and the number of iterations is 500. The crossover operator and mutation operator are determined by the improved method in this paper. We use MATLAB to calculates the optimized fusion estimation value. To avoid contingency, this paper conducted 100 experiments, taking an average of 100 sets of operation results, and plotting the improved fitness (objective function) curve after optimization. The results are shown in Figure 4.

The optimal estimate after 500 iterations $\hat{Y}$= 0.8172 and the error between the real value and the optimal estimate can be obtained by using Formula (12) the ρ = 0.0043. It can also be seen from Figure 4 that, after number is 50 times iterative evolutions, the average fitness and the maximum fitness of the population have mutually similar patterns, indicating that the convergence of the algorithm proceeds smoothly and there is no oscillation. Under the premise, individuals with the greatest fitness have not evolved for several consecutive generations, indicating that the population has matured and reached the evolutionary requirements.

### 6.4. Performance Comparison

**Figure 5:**
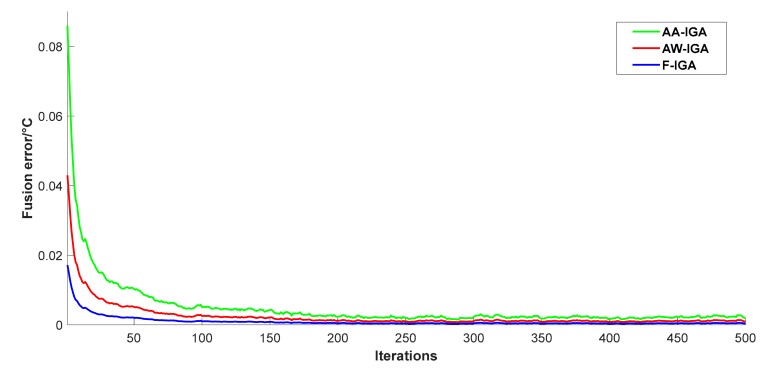
Fusion error curve obtained by three algorithms.

In order to analyze and compare the performance of different fusion algorithms, this paper examines and analyzes the fusion error and execution time. The genetic algorithm improved by this paper is used to optimize the fusion results of two commonly used fusion algorithms, arithmetic average method, and adaptive weighting method. The initial population size and the number of iterations are the same as those set previously. One hundred trials as before, taking the average and the fusion errors of the three algorithms are calculated, and the curve is drawn, as shown in Figure 5.

After 500 iterations, the fusion errors based on Trust degree Improved Genetic Algorithm (F-IGA), Arithmetic Mean Improved Genetic Algorithm (AA-IGA), and Adaptive Weighting Improved Genetic Algorithm (AW-IGA) are available. See Table 1.

From Table 1, we can see that the fusion errors of the three algorithms F-IGA, AA-IGA, and AW-IGA are 0.0043, 0.0107, and 0.0076, respectively. The fusion accuracy based on the F-IGA algorithm is 2.49 times that of the AA-IGA algorithm and 1.78 times that of the AW-IGA algorithm. It can be seen that, with the data fusion algorithm based on trust degree-improved genetics proposed in this paper, the fusion error is significantly reduced, which effectively improves the fusion precision and system stability.

MATLAB’s profiler using to calculate the average running time of each of the three algorithms running 100 times, the results are shown in Table 2.

It can be seen from Table 2 that the average running time of the F-IGA algorithm is 64.63% shorter than that of the AA-IGA algorithm, which is 54.24% shorter than the AW-IGA algorithm, which greatly improved the performance of the algorithm and effectively reduces the energy consumption of the sensor node, extends sensor life.

## 7. Conclusions

Aiming at the problem of low precision and poor stability of multi-sensor data fusion, this paper proposes a data fusion algorithm based on trust degree and improved genetics, and deploys WSN system in a modern greenhouse environment for field test. The most apparent findings to emerge from this study is that: (1) Three-index smoothing can effectively reduce data fluctuations and improve the stability of the greenhouse WSNs system. (2) Compared with the arithmetic average and adaptive weighted data fusion algorithms, the data fusion algorithm based on trust degree and improved genetics has a significantly shorter average running time and higher data fusion precision. In summary, the data fusion algorithm proposed in this paper has good applicability and can meet the requirements of data fusion of greenhouse WSNs system.

## Figures and Tables

**Table 1:** Fusion error of three algorithms.

Algorithm	F-IGA	AA-IGA	AW-IGA
Fusion error (°C)	0.0043	0.0107	0.0076

**Table 2:** The average running time of the three algorithms.

Algorithm	F-IGA	AA-IGA	AW-IGA
Average running time (s)	21.274	60.155	46.491
